# Interplanar coupling-dependent magnetoresistivity in high-purity layered metals

**DOI:** 10.1038/ncomms10903

**Published:** 2016-03-29

**Authors:** N. Kikugawa, P. Goswami, A. Kiswandhi, E. S. Choi, D. Graf, R. E. Baumbach, J. S. Brooks, K. Sugii, Y. Iida, M. Nishio, S. Uji, T. Terashima, P.M.C. Rourke, N. E. Hussey, H. Takatsu, S. Yonezawa, Y. Maeno, L. Balicas

**Affiliations:** 1 National Institute for Materials Science, Tsukuba, Ibaraki 305-0003, Japan; 2 Condensed Matter Group, National High Magnetic Field Laboratory, Florida State University, 1800 East Paul Dirac Drive, Tallahassee, Florida 32310, USA; 3 Condensed Matter Theory Center, University of Maryland, College Park, Maryland 20742-4111, USA; 4 National Institute for Materials Science, Tsukuba, Ibaraki 305-0047, Japan; 5 H.H. Wills Physics Laboratory, University of Bristol, Tyndall Avenue, Bristol BS8 1TL, UK; 6 High Field Magnet Laboratory (HFML-EMFL), Radboud University, Toernooiveld 7, 6525 ED Nijmegen, Nijmegen, The Netherlands; 7 Institute of Molecules and Materials, Radboud University, Heyendaalseweg 135, 6525 AJ Nijmegen, The Netherlands; 8 Department of Physics, Tokyo Metropolitan University, Tokyo 192-0397, Japan; 9 Department of Physics, Graduate School of Science, Kyoto University, Kyoto 606-8502, Japan

## Abstract

The magnetic field-induced changes in the conductivity of metals are the subject of intense interest, both for revealing new phenomena and as a valuable tool for determining their Fermi surface. Here we report a hitherto unobserved magnetoresistive effect in ultra-clean layered metals, namely a negative longitudinal magnetoresistance that is capable of overcoming their very pronounced orbital one. This effect is correlated with the interlayer coupling disappearing for fields applied along the so-called Yamaji angles where the interlayer coupling vanishes. Therefore, it is intrinsically associated with the Fermi points in the field-induced quasi-one-dimensional electronic dispersion, implying that it results from the axial anomaly among these Fermi points. In its original formulation, the anomaly is predicted to violate separate number conservation laws for left- and right-handed chiral (for example, Weyl) fermions. Its observation in PdCoO_2_, PtCoO_2_ and Sr_2_RuO_4_ suggests that the anomaly affects the transport of clean conductors, in particular near the quantum limit.

## Abstract (web summary)

In Weyl semimetals, unusual electronic transport phenomena are predicted to occur, such as an axial anomaly which violates the conservation of chiral fermions. Here, the authors evidence such behaviour via the occurrence of negative magnetoresistance in layered high-purity non-magnetic metals.

## Graphical abstract

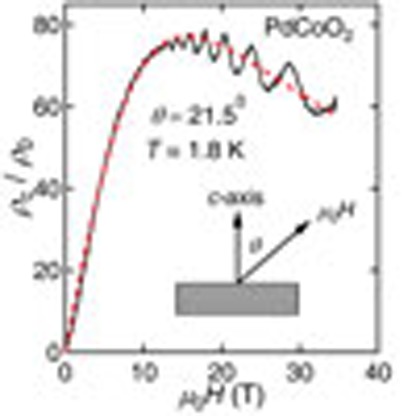

The magnetoconductivity or -resistivity of metals under a uniform magnetic field *μ*_0_*H* (*μ*_0_ is the permeability of free space) is highly dependent on the precise shape of their Fermi surface (FS) and on the orientation of the current flow relative to the external applied field *H*^1^,^2^. This is particularly true for high-purity metals at low temperatures, whose carriers may execute many cyclotronic orbits in between scattering events. However, the description of the magnetoconductivity of real systems in terms of the Boltzmann equation including the Lorentz force, the electronic dispersion and realistic scattering potentials is an incredibly daunting task, whose approximate solutions can only be obtained through over simplifications. Despite the inherent difficulty in describing the magnetoresistivity of metallic or semi-metallic systems, it continues to be a subject of intense interest. Indeed, in recent years, a number of new magnetoresistance phenomena have been uncovered. For example, although semi-classical transport theory predicts a magnetoresistivity *ρ*(*μ*_0_*H*)∝(*μ*_0_*H*)^2^, certain compounds such as *β*-Ag_2_Te display a linear, non-saturating magnetoresistivity^3^, which is ascribed to the quantum magnetoresistive scenario^4^, associated with linearly dispersing Dirac-like bands^5^. However, in semi-metals characterized by a bulk Dirac dispersion and extremely high electron mobilities such as Cd_3_As_2_, the linear magnetoresistivity develops a weak (*μ*_0_*H*)^2^ term as the quality of the sample increases^6^. Its enormous magnetoresistivity is claimed to result from the suppression of a certain protection against backscattering channels^6^. The semi-metal WTe_2_ was also found to display a very large and non-saturating magnetoresistivity, which is ∝(*μ*_0_*H*)^2^ under fields up to 60 T. This behaviour was ascribed to a nearly perfect compensation between the densities of electrons and holes^7^. In recent times, a series of compounds were proposed to be candidate Weyl semi-metals characterized by a linear touching between the valence and the conduction bands at several points (Weyl points) of their Brillouin zone^8^. These Weyl points are predicted to lead to a pronounced negative magnetoresistivity for electric fields aligned along a magnetic field due to the so-called axial anomaly^9^,^10^.

Here we unveil the observation of yet another magnetoresistive effect, namely a pronounced negative magnetoresistivity in extremely clean and non-magnetic layered metals. We study the delafossite-type PtCoO_2_ and PdCoO_2_ compounds, which are characterized by a single FS sheet and, as with Cd_3_As_2_, can display residual resistivities on the order of a just few tenths of nΩ cm. Given its extremely low level of disorder, for specific field orientations along which the interlayer coupling vanishes, PdCoO_2_ can display a very pronounced positive magnetoresistivity that exceeds 550,000% for *μ*_0_*H*≃35 T and for currents along the interlayer axis. Nevertheless, as soon as the field is rotated away from these specific orientations and as the field increases, this large orbital effect is overwhelmed by the emergence of a pronounced negative magnetoresistivity. For fields along the interlayer direction, a strong longitudinal negative magnetoresistivity is observed from *μ*_0_*H*=0 T to fields all the way up to *μ*_0_*H*=35 T. Very similar behaviour is observed in the PtCoO_2_ compound. For the correlated Sr_2_RuO_4_, the longitudinal negative magnetoresistivity effect is also observable but only in the cleanest samples, that is, those displaying the highest superconducting transition temperatures. We suggest that this effect might result from the axial anomaly between Fermi points in a field-induced, quasi-one-dimensional electronic dispersion.

## Results

### Observation of an anomalous longitudinal magnetoresistivity

**Figure 1:**
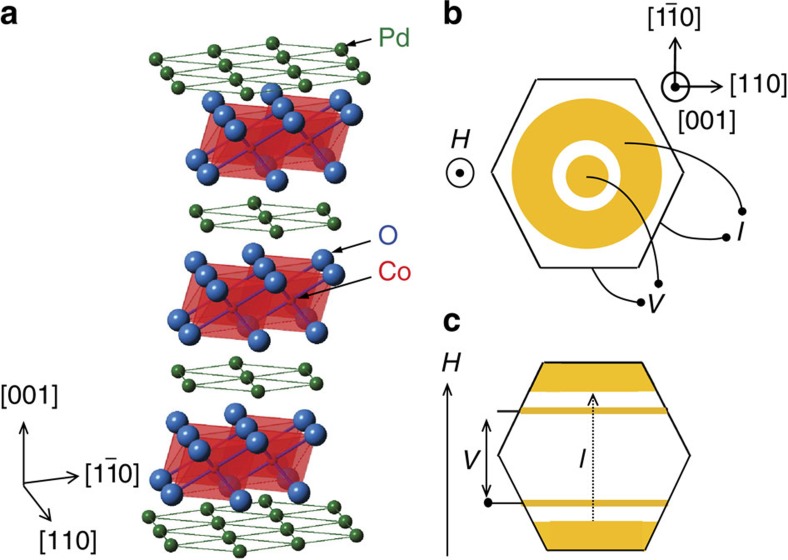
Crystal structure of PdCoO_2_ and configuration of electrical contacts. (**a**) Crystallographic structure of the delafossite PdCoO_2_ with Pd, Co and O atoms shown in green, blue and red, respectively. (**b**) Configuration of contacts for measuring the interplanar longitudinal resistivity (*ρ*_c_), showing concentric contacts at the top and at the bottom surface of each hexagonal platelet-like crystal. (**c**) Configuration of contacts for measuring the in-plane longitudinal resistivity 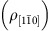 for currents flowing along the 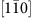 axis and fields applied along the same direction.

As shown in Fig. 1a, PdCoO_2_ crystallizes in the space group 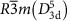, which results from the stacking of monatomic triangular layers^11^. The synthesis of PdCoO_2_ single crystals is described in the Methods section. According to band structure calculations^12^,^13^,^14^, the Fermi level *E*_F_ is placed between the filled *t*_2g_ and the empty *e*_g_ levels with the Pd triangular planes dominating the conductivity and leading to its highly anisotropic transport properties. The reported room temperature in-plane resistivity is just 2.6 μΩ cm, making PdCoO_2_ perhaps the most conductive oxide known to date^15^. Figure 1b,c show the configuration of contacts used for measuring the longitudinal magnetoresistivity of all compounds. de Haas van Alphen measurements^15^ reveal a single, corrugated and nearly two-dimensional FS with a rounded hexagonal cross-section, in broad agreement with both band structure calculations^12^,^13^,^14^ and angle-resolved photoemission measurements^16^. de Haas van Alphen yields an average Fermi wave vector 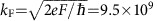 m^−1^or an average Fermi velocity 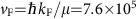 m s^−1^ (where *μ*≃1.5 is the carrier effective mass^15^ in units of free electron mass). Recent measurements of interplanar magnetoresistivity *ρ*_c_(*μ*_0_*H*) reveal an enormous enhancement for fields along the 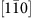 direction, that is, increasing by ∼35,000% at 2 K under *μ*_0_*H*=14 T, which does not follow the characteristic *ρ*(*μ*_0_*H*)∝(*μ*_0_*H*)^2^ dependence at higher fields^17^. This behaviour can be reproduced qualitatively by semi-classical calculations, assuming a very small scattering rate^17^. Most single crystals display in-plane residual resistivities *ρ*_ab0_ ranging from only ∼10 up to ∼40 nΩ cm, which correspond to transport lifetimes 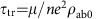 ranging from ⪞20 down to ≃5.5 ps (*e* is the electron charge and *n*≃2.4 × 10^28^ m^−3^ (ref. 11)) or mean free paths 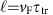 ranging from ∼4 up to 20 μm (ref. 15). However, according to ref. 15, the quasiparticle lifetime 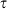 extracted from the Dingle temperature becomes (in units of length) 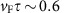 μm. Hence, the transport lifetime is larger than the quasiparticle lifetime by at least one order of magnitude, which is the hallmark of a predominant forward scattering mechanism (see ref. 18). For a magnetic field along *c* axis, 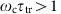 when *μ*_0_*H*⪞1 T; in contrast, 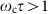 when *μ*_0_*H*>10 T. These estimations suggest the importance of the Landau quantization for understanding our observations over a wide range of fields up to *μ*_0_*H*∼30 T.

**Figure 2:**
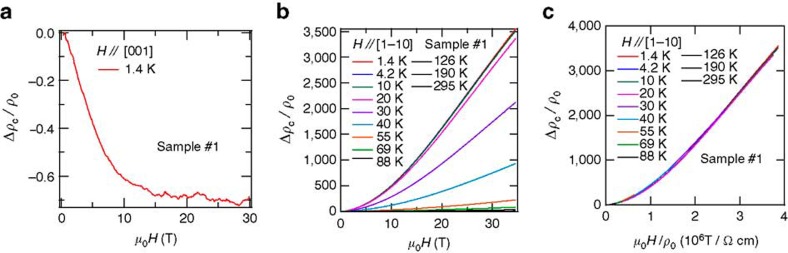
Negative longitudinal and colossal orbital magnetoresistance of PdCoO_2_. (**a**) Normalized interplanar magnetoresistivity Δ*ρ*_c_/*ρ*_0_=(*ρ*_c_(*μ_0_H*)−*ρ*_0_)/*ρ*_0_, where *ρ*_0_ is the resistivity at zero field, for a PdCoO_2_ single crystal and as a function of 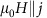 axis at *T*=1.4 K. The very pronounced negative longitudinal magnetoresistance arising in the presence of cyclotron motion in the *ab* plane is noteworthy. (**b**) Δ*ρ*_c_(*μ_0_H*)/*ρ*_0_ as a function of *μ_0_H* applied along the 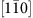 direction and for several temperatures *T*, describing positive transverse magnetoresistance. At *T*=1.4 K, Δ*ρ*_c_ surpasses 350,000% under a field *H*=35 T. (**c**) Kohler scaling of the transverse positive magnetoresistance Δ*ρ*_c_(*μ_0_H*). It is noteworthy that (i) all data collapse on a single curve as a function of *μ*_0_*H*/*ρ*_0_ and (ii) at low fields Δ*ρ*_c_(*μ_0_H*)/*ρ*_0_∝(*μ_0_H*/*ρ*_0_)^2^ as expected for classical orbital magnetoresistance.

As shown in Fig. 2a, the low-*T* magnetoresistivity or Δ*ρ*_c_=(*ρ*_c_−*ρ*_0_)/*ρ*_0_, where *ρ*_0_ is the zero-field interplanar resistivity, decreases (up to ∼70%) in a magnetic field of 30 T oriented parallel to the applied current. Given that PdCoO_2_ is non-magnetic and extremely clean (see Methods), this effect cannot be attributed to magnetic impurities. In addition, the magnitude of the observed magnetoresistivity cannot be explained in terms of weak localization effects^19^,^20^. To support both statements, in Fig. 2b we show Δ*ρ*_c_ for a PdCoO_2_ single crystal as a function of *H* applied along the 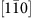 planar direction and for several temperatures *T*. In sharp contrast to results shown in Fig. 2a, as *T* decreases, Δ*ρ*_c_(*μ*_0_*H*) increases considerably, by more than three orders of magnitude when *T*<10 K, thus confirming the absence of scattering by magnetic impurities or any role for weak localization. In addition, it is noteworthy that Δ*ρ*_c_∝(*μ*_0_*H*)^2^ at low fields, which indicates that the interlayer transport is coherent at low fields^21^. Figure 2c depicts a simple Kohler plot of the magnetoresistivity shown in Fig. 2b, where the field has also been normalized by *ρ*_0_(*T*), which indicates unambiguously that the transverse magnetoresistive effect in PdCoO_2_ is exclusively orbital in character and is dominated by the scattering from impurities/imperfections and phonons^1^.

**Figure 3:**
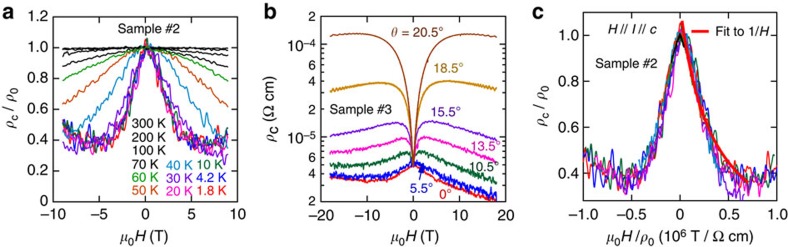
Anomalous magnetoresistive response of PdCoO_2_. (**a**) Interlayer resistivity *ρ*_c_ normalized by its zero-field value *ρ*_0_ as a function of the external field *μ*_0_*H* and for *μ*_0_*H* parallel to current *I* (itself parallel to the sample interlayer *c*-axis) and for several temperatures *T*. It is noteworthy that the very pronounced negative magnetoresistivity, that is, *ρ*_c_/*ρ*_0_ decreases by a factor >60% when sweeping the field from 0 to 5 T. It is also worth noting that this effect disappears when the *T* approaches and/or surpasses ∼200 K. (**b**) *ρ*_c_ as a function of *μ_0_H* from a third crystal at *T*=1.8 K and for several angles *θ* between *μ*_0_*H* and the *c* axis. It is noteworthy how the negative magnetoresistivity observed at low fields is progressively suppressed as *θ* increases, becoming strongly positive. Nevertheless, the mechanism leading to the negative magnetoresistivity is observed to overpower the orbital one at higher fields and higher angles. (**c**) Kohler plot for all the temperature-dependent *ρ*_c_/*ρ*_0_. Red line is a fit of Δ*ρ*_c_/*ρ*_0_ to (*μ*_0_*H*)^−1^.

The evolution of the longitudinal magnetoresistance with temperature is depicted in Fig. 3a. *ρ*_c_ is seen to decrease by a factor surpassing 60% for fields approaching 9 T and for all temperatures below 30 K. Figure 3b displays *ρ*_c_(*μ*_0_*H*)/*ρ*_0_ as a function of the angle *θ* between *μ*_0_*H* and the *c* axis at a temperature *T*=1.8 K, for a third single crystal. For *θ*>10°, the pronounced positive magnetoresistance observed at low fields, due to an orbital magnetoresistive effect, is overpowered at higher fields by the mechanism responsible for the negative magnetoresistivity. This behaviour is no longer observed within this field range when *θ* is increased beyond ∼20°. Figure 3c shows a Kohler plot, that is, Δ*ρ*_c_/*ρ*_0_ as a function of *μ*_0_*H* normalized by *ρ*_0_. As seen in Fig. 3c, all curves collapse on a single curve, indicating that a particular transport mechanism dominates even at high temperatures where phonon scattering is expected to be strong. The red line is a fit to (*μ*_0_*H*)^−1^, indicating that 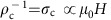 at lower fields.

### Angular dependence of the anomalous magnetoresistive response

**Figure 4:**
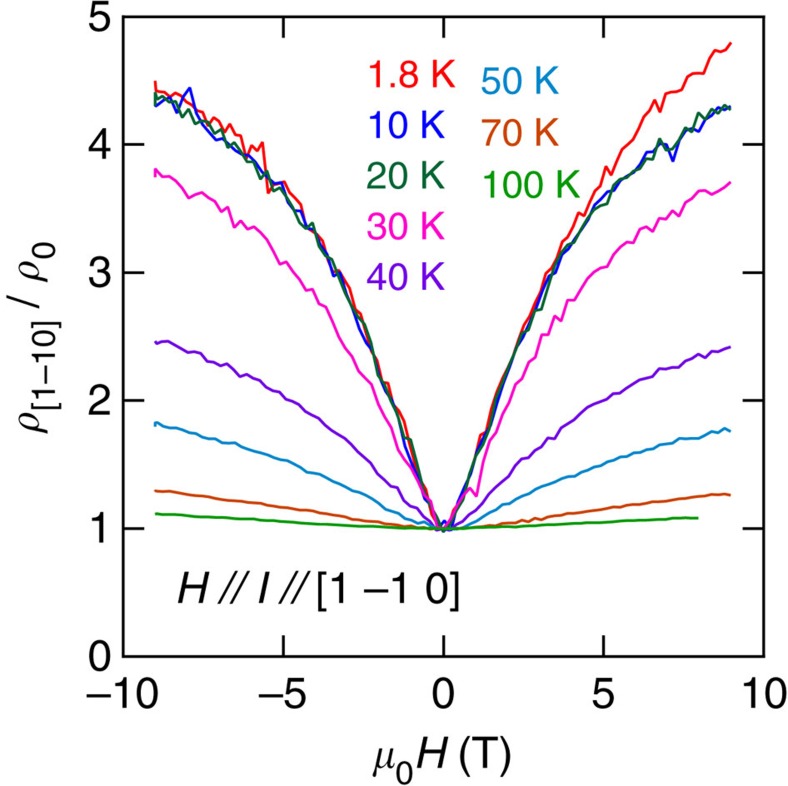
Longitudinal magnetoresistance for fields along the planes. In-plane longitudinal resistivity 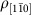 normalized by its zero field value *ρ*_0_ as a function of the field applied along the 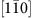 direction, for a PdCoO_2_ single crystal and for several temperatures. The absence of negative magnetoresistivity is noteworthy.

Fig. 4 shows the longitudinal magnetoresistance 
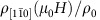
 for fields and currents along the 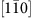 axis. For this orientation, the charge carriers follow open orbits along the axis of the cylindrical FS instead of quantized cyclotronic orbits. In contrast to Δ*ρ*_c_/*ρ*_0_, but similar to the longitudinal magnetoresistivity of ultra-clean elemental metals^1^,^2^, 
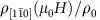
 is observed to increase and saturate as a function of *μ*_0_*H*. This further confirms that conventional mechanisms, for example, impurities, magnetism and so on, are not responsible for the negative longitudinal magnetoresistivity observed in Δ*ρ*_c_/*ρ*_0_.

**Figure 5:**
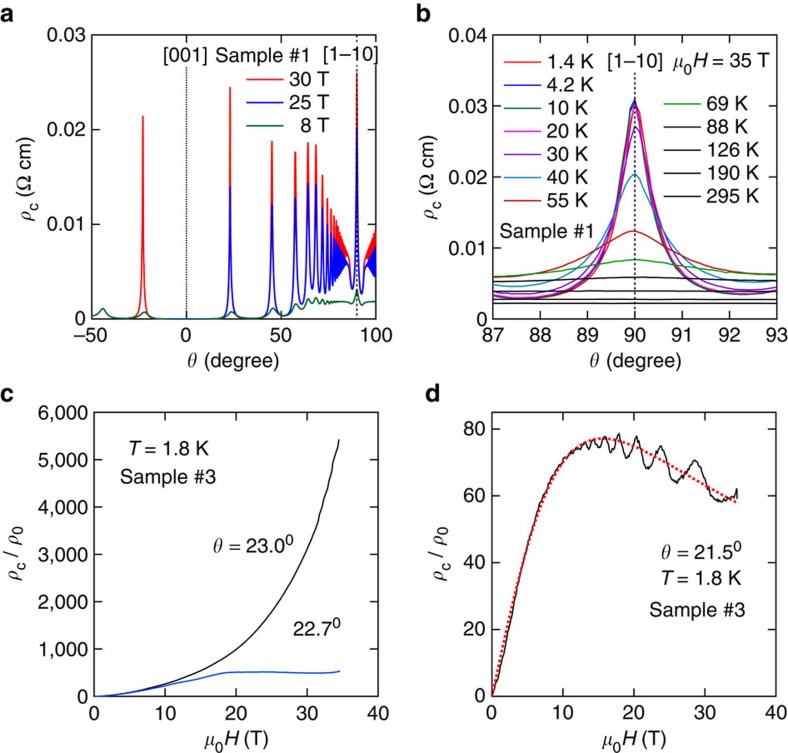
Angular magnetoresistance oscillations for a PdCoO_2_ single crystal. (**a**) Interplanar magnetoresistivity *ρ*_c_ for a PdCoO_2_ single crystal as a function of the angle *θ* between the [001] interplanar direction and the external field *μ_0_H*. The pronounced peaks observed as a function of *θ* are the so-called Yamaji-effect peaks^22^. (**b**) Interlayer coherence peak observed for fields nearly along the interplanar direction, which indicates an extended FS along the interlayer direction^24^. From the width Δ*θ* of the peak at half maximum, one can estimate the value of the interlayer transfer integral *t*_c_=2.79 meV from equation (1). (**c**) Interplanar resistivity *ρ*_c_ as a function of *μ_0_H* at *T*=1.8 K and for two angles, that is, the Yamaji value *θ*_*n*=1_=23.0° and *θ*=22.7°. It is noteworthy how the pronounced positive magnetoresistivity observed at *θ*_*n*=1_ is strongly suppressed when *μ_0_H* is rotated by just ∼0.3°, leading to magnetoresistance saturation. (**d**) *ρ*_c_ as a function of *μ*_0_*H* under *T*=1.8 K and for *θ*=21.5°. It is noteworthy how *ρ*_c_, after increasing by several orders of magnitude, displays negative magnetoresistivity at higher fields, thus indicating a clear competition between the orbital and another mechanism, which suppresses the magnetoresistivity. Dotted red line corresponds to a fit of 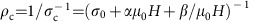.

Figure 5a shows *ρ*_c_ as a function of the angle *θ* between the field and the *c* axis, for three different field values: 8, 25 and 30 T. *ρ*_c_(*θ*) displays the characteristic structure displayed by quasi-two-dimensional metals, namely a series of sharp peaks at specific angles 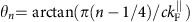 called the Yamaji angles (where *n* is an integer, *c* is the interplanar distance and 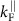 is the projection of the Fermi wave number on the conduction plane), for which all cyclotronic orbits on the FS have an identical orbital area^22^. In other words, the corrugation of the FS no longer leads to a distribution of cross-sectional areas, as if the corrugation has been effectively suppressed. As discussed below, in terms of the energy spectrum, this means that the Landau levels become non-dispersive at the Yamaji angles^18^,^23^; hence, one no longer has Fermi points. The sharp peak at *θ*=90° is attributed to coherent electron transport along small closed orbits on the sides of a corrugated cylindrical FS^24^,^25^. The width of this peak Δ*θ*, shown in Fig. 5b for several temperatures, allows us to estimate the interlayer transfer integral *t*_c_ (ref. 26),


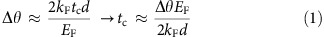


assuming a simple sinusoidal FS corrugation along the *k*_z_ direction. Here, the interplanar separation is *d*=*c*/3, as there are three conducting Pd planes per unit cell, each providing one conducting hole and therefore leading to three carriers per unit cell. This value is consistent with our Hall-effect measurements (not included here). The full width at half maximum of the peak at 90° is Δ*θ*≃0.78° and *E*_F_ is given by 
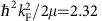
 eV; therefore, one obtains *t*_c_=2.79 meV or ≃32.4 K. Figure 5c displays *ρ*_c_ as a function of *μ*_0_*H* for two angles; the Yamaji angle *θ*_*n*=1_=23.0° and *θ*=22.7°, respectively. As seen, *ρ*_c_(*μ*_0_*H*) for fields along *θ*_*n*=1_ displays a very pronounced positive magnetoresistance, that is, *ρ*_c_/*ρ*_0_ increases by ∼550,000% when *μ*_0_*H* is swept from 0 to 35 T. However, at *μ*_0_*H*=35 T, *ρ*_c_/*ρ*_0_ decreases by one order of magnitude as *μ*_0_*H* is rotated by just ∼0.3° with respect to *θ*_*n*=1_. Furthermore, as seen in Fig. 5d, at higher fields *ρ*_c_ displays a cross-over from a very pronounced and positive to a negative magnetoresistance, resulting from a small increment in *θ* relative to *θ*_*n*=1_. This is a very clear indication for two competing mechanisms, with negative magnetoresistivity overcoming the orbital effect when the orbitally averaged interlayer group velocity (or the transfer integral *t*_c_) becomes finite at *θ*≠*θ*_*n*_. We emphasize that for a conventional and very clean metal, composed of a single FS sheet, the magnetoresistivity should either be ∝(*μ*_0_*H*)^2^ (ref. 21) or saturate as seen in quasi-two-dimensional metals close to the Yamaji angle^27^, or in Fig. 2a,b for fields below ∼15 T. This is illustrated by the Supplementary Fig. 1 (see also Supplementary Note 1), which contrasts our experimental observations with predictions based on semi-classical transport models, which correctly describe the magnetoresistance of layered organic metals in the vicinity of the Yamaji angle. In contrast, as illustrated by the dotted red line in Fig. 5d, *ρ*_c_(*μ*_0_*H*) can be well described by the expression 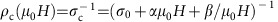. Here, the *ρ*_c_∝(*μ*_0_*H*)^−1^ term describes the negative magnetoresistivity as previously seen in Fig. 3, whereas the *ρ*_c_∝*μ*_0_*H* term describes the non-saturating linear magnetoresistance predicted and observed for systems close to the quantum limit^3^,^4^,^5^,^28^. This expression describes *ρ*_c_(*μ*_0_*H*, *θ*) satisfactorily, except at the Yamaji angle where both terms vanish. In the neighbourhood of *θ*_*n*_, the addition of a small *ρ*_c_∝(*μ*_0_*H*)^2^ term improves the fit, with its pre-factor increasing as *θ*_*n*_ is approached. *ρ*_c_ also displays Shubnikov de Haas oscillations at small (and strongly *θ* dependent) frequencies, which were not previously detected in ref. 15. As discussed in ref. 29, these slow oscillations, observed only in the interlayer magnetoresistance of layered metals, originate from the warping of the FS. In Supplementary Fig. 2 (See also Supplementary Note 2), we show how these frequencies disappear when the group velocity vanishes at *θ*_*n*_.

**Figure 6:**
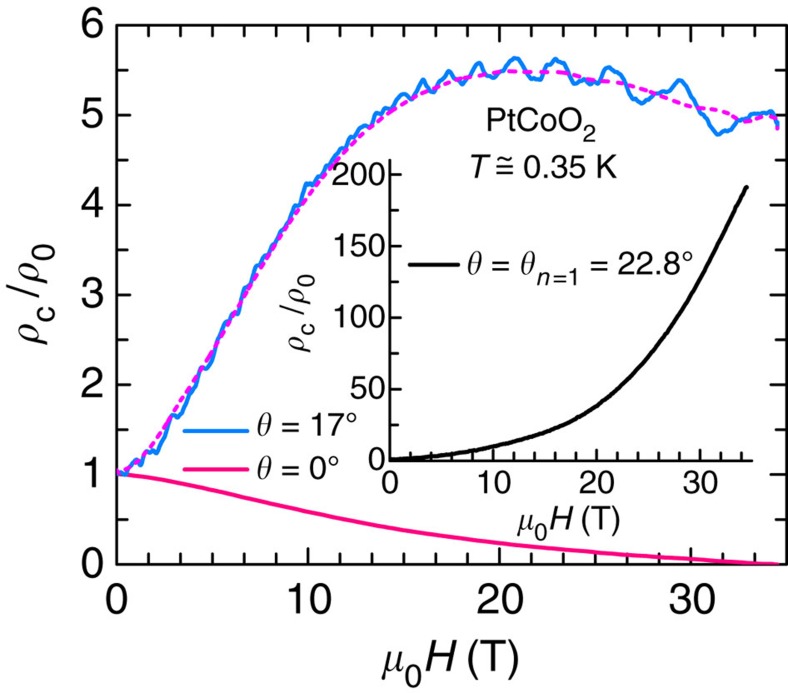
Negative longitudinal magnetoresistance in PtCoO_2_. Interplanar resistivity *ρ*_c_ normalized by its zero-field value *ρ*_0_ for a PtCoO_2_ single crystal at a temperature *T*=0.35 K and as a function of the magnetic field *μ*_0_*H* applied along two angles with respect to the *c* axis, respectively *θ*=0° (pink line) and 17° (blue line). Dashed magenta line describes the smoothly varying background. Inset: *ρ*_c_/*ρ*_0_ as a function of *μ*_0_*H* applied along the first Yamaji angle *θ*_*n*_=22.8°.

Significantly, this effect does not appear to be confined to PdCoO_2_. Figure 6 presents an overall evaluation of the longitudinal magnetoresistance of isostructural PtCoO_2_, whereas Supplementary Fig. 3 displays the observation of impurity-dependent negative magnetoresistivity in the correlated perovskite Sr_2_RuO_4_ (See also Supplementary Note 3). As shown in Fig. 6, PtCoO_2_ presents a pronounced negative longitudinal magnetoresistivity either for 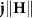 *c* axis or for *μ*_0_*H* close to an Yamaji angle (**j** is the current density). It also presents a very pronounced and non-saturating magnetoresistivy for fields applied along the Yamaji angle. For both systems, the magnetoresistivity does not follow a single power law as a function of *μ*_0_*H*. In fact, as shown in Supplementary Fig. 4, at *θ*_*n*_ the magnetoresistivity of the (Pt,Pd)CoO_2_ system follows a (*μ*_0_*H*)^2^ dependence for *μ*_0_*H*≲15 T. At intermediate fields, *ρ*(*μ*_0_*H*) deviates from the quadratic dependence, recovering it again at subsequently higher fields. As Kohler's rule implies that Δ*ρ*/*ρ*_0_∝(*μ*_0_*H*/*ρ*_0_)^2^, we argue that the observed increase in slope would imply a field-dependent reduction in scattering by impurities (see Supplementary Fig. 4 and Supplementary Note 4). The precise origin of this suppression in scattering remains to be identified. Nevertheless, the enormous and positive magnetoresistivity observed for fields along *θ*_*n*_ seems consistent with a simple scenario, that is, an extremely clean system(s) whose impurity scattering weakens with increasing magnetic field. In Sr_2_RuO_4_, the negative longitudinal magnetoresistivity is observed only in the cleanest samples and for angles within 10° away from the *c* axis. This compound is characterized by three corrugated cylindrical FS sheets, each leading to a distinct set of Yamaji angles, making it impossible to completely suppress the interplanar coupling at specific Yamaji angle(s).

## Discussion

Negative magnetoresistivity is a common feature of ferromagnetic metals near their Curie temperature, or of samples having dimensions comparable to their electronic mean free path where the winding of the electronic orbits under a magnetic field reduces the scattering from the surface. It can also result from the field-induced suppression of weak localization or from the field-induced suppression of spin-scattering/quantum-fluctuations as seen in *f*-electron compounds^30^. None of the compounds described in this study are near a magnetic instability, nor do they contain significant amounts of magnetic impurities or disorder to make them prone to weak localization. The magnitude of this anomalous magnetoresistivity, coupled to its peculiar angular dependence, are in fact enough evidence against any of these conventional mechanisms. Below, we discuss an alternative scenario based on the axial anomaly, which in our opinion explains most of our observations.

The axial anomaly is a fundamental concept of relativistic quantum field theory, which describes the violation of separate number conservation laws of left- and right-handed massless chiral fermions in odd spatial dimensions due to quantum mechanical effects^31^,^32^. When three-dimensional massless Dirac or Weyl fermions are placed under parallel electric and magnetic fields, the number difference between the left and the right-handed fermions is expected to vary with time according to the Adler–Bell–Jackiw formula^9^,^33^


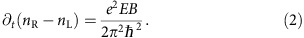


Here, *n*_R/L_ are the number operators for the right- and the left-handed Weyl fermions, with the electric and the magnetic field strengths respectively given by *E* and *B*. The Dirac fermion describes the linear touching of twofold Kramers degenerate conduction and valence bands at isolated momentum points in the Brillouin zone. By contrast, the Weyl fermions arise due to the linear touching between nondegenerate conduction and valence bands. The axial anomaly was initially proposed to produce a large, negative longitudinal magnetoresistance, for a class of gapless semiconductors, for which the low-energy band structure is described by massless Weyl fermions^10^. The reason for the negative magnetoresistance is relatively straightforward. The number imbalance due to axial anomaly can only be equilibrated through backscattering between two Weyl points. This involves a large momentum transfer **Q**_W_. Quite generally the impurity scattering in a material can be modeled by a momentum dependent impurity potential *V*(**Q**), where **Q** is the momentum transfer between the initial and the final electronic states. If *V*(**Q**) is a smoothly decreasing function of |**Q**| (such as Gaussian or Lorentzian), the backscattering amplitude can be considerably smaller than its forward scattering counterparts (occurring with small **Q** around each Weyl point). Therefore in the presence of axial anomaly the transport lifetime can be considerably larger than the one in the absence of a magnetic field. Consequently the axial anomaly in the presence of parallel **E** and **B** fields can give rise to larger conductivity or smaller resistivity i.e., negative magnetoresistance. Recent theoretical proposals for Weyl semi-metals^34^,^35^,^36^,^37^ followed by experimental confirmation^38^,^39^ have revived the interest in the experimental confirmation of the axial anomaly through efforts in detecting negative longitudinal magnetoresistivity^40^,^41^,^42^,^43^,^44^,^45^,^46^. There are examples of three-dimensional Dirac semi-metals^47^,^48^,^49^, which may be converted, through Zeeman splitting, into a Weyl semi-metal. Examples include Bi_1−*x*_Sb_*x*_ at the band inversion transition point between topologically trivial and nontrivial insulators^42^, and Cd_3_As_2_ (ref. 6).

**Figure 7:**
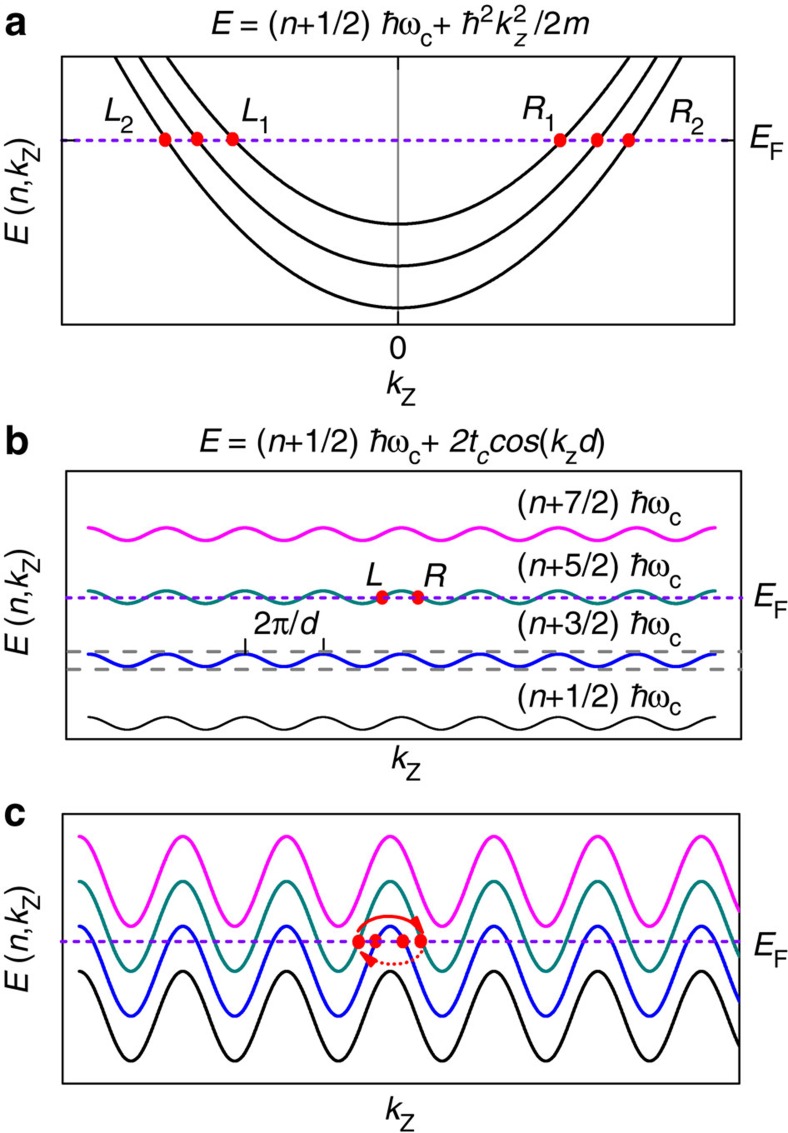
Field-induced electronic dispersion for metals of different dimensionality. (**a**) The dispersion of Landau levels for a conventional three-dimensional metal placed in an external magnetic field applied along the *z*-direction. Owing to the underlying parabolic dispersion, each Landau level disperses quadratically as a function of *k*_z_, the momentum component along the applied field. Each partially occupied Landau level intersects the Fermi energy *E*_F_ at two Fermi points, as indicated by the red dots. In the vicinity of the two Fermi points located at *k*_z_=±*k*_F,n_ for the *n*-th partially filled Landau level, the quasiparticles disperse linearly with opposite group velocities *v*_±,n_=±*ħk*_F,n_/*μ* where *μ* is the effective mass. The ± signs of the group velocity respectively define the chirality of the right- and the left-moving one-dimensional fermions. (**b**) In contrast, for quasi-two-dimensional metals the Landau levels possess a periodic dispersion relation as a function of *k*_z_, owing to the tight binding term 2*t*_c_ cos(*k*_z_*d*), with interlayer hopping strength and spacing, respectively, given by *t*_c_ and *d*. Within the first Brillouin zone defined as −*π*/*d*<*k*_z_<*π*/*d*, each partially filled Landau level again gives rise to a pair of one-dimensional fermions of opposite chirality around the Fermi points. The situation depicted here corresponds to 4*t*_c_<*ħω*_c_, or when only one Landau level is partially filled. (**c**) Landau levels for 4*t*_c_>*ħω*_c_ or when multiple Landau levels are partially occupied and each of them gives rise to a pair of chiral fermions.

In analogy with the predictions for the axial anomaly between Weyl points, here we suggest that our observations might be consistent with the emergence of the axial anomaly among the Fermi points of a field-induced, one-dimensional electronic dispersion^18^. In effect, in the presence of a strong magnetic field, the quantization of cyclotron motion leads to discrete Landau levels with one-dimensional dispersion and a degeneracy factor *eB*/*h*, see Fig. 7a–c. Consider the low-energy description of a one-dimensional electron gas, in terms of the right- and left-handed fermions obtained in the vicinity of the two Fermi points. In the presence of an external electric field *E*, the separate number conservation of these chiral fermions is violated according to


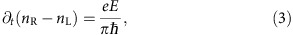


where *n*_R/L_ corresponds to the number operators of the right- and left-handed fermions, respectively^31^,^32^. Each partially occupied Landau level leads to a set of Fermi points and the axial anomaly for such a level can be obtained from equation 3, after multiplying by *eB*/*h*. Therefore, each level has an axial anomaly determined by equation (2). When only one Landau level is partially filled, we have the remarkable universal result for the axial anomaly described by Adler–Bell–Jackiw formula of equation (2). For a non-relativistic electron gas, this would occur at the quantum limit. In contrast, this situation would naturally occur for Dirac/Weyl semi-metals, when the Fermi level lies at zero energy, that is, the material has a zero carrier density. Figure 7b describes the situation for a quasi-two-dimensional electronic system on approaching the quantum limit, or when the interplanar coupling becomes considerably smaller than the inter Landau level separation (for example, in the vicinity of the Yamaji angle). We emphasize that the observation of a pronounced, linear-in-field magnetoresistive component, as indicated by the fit in Fig. 5d, is a strong experimental evidence for the proximity of PdCoO_2_ to the quantum limit on approaching the Yamaji angle. Therefore, we conclude that the axial anomaly should be present in every three-dimensional conducting system, on approaching the quantum limit. Explicit calculations indicate that the axial anomaly would only cause negative magnetoresistance for predominant forward scattering produced by ionic impurities^18^,^50^. *ρ*(*μ*_0_*H*)∝(*μ*_0_*H*)^−1^ as observed here (Figs 3 and 5) would result from Gaussian impurities^18^. As our experimental results show, PdCoO_2_ is a metal of extremely high conductivity, thus necessarily dominated by small-angle scattering processes and therefore satisfying the forward scattering criterion. In this metal the Landau levels disperse periodically as shown in Fig. 7b,c, depending on the relative strength of the cyclotron energy *ħω*_c_=*ħeB*/*μ* with respect to the interlayer transfer integral *t*_c_. The condition 4*t*_c_>*ħω*_c_ is satisfied when *μ*_0_*H* roughly exceeds 100 T. For this reason, Fig. 7c, with multiple partially occupied Landau levels, describes PdCoO_2_ for fields along the *c* axis or for arbitrary angles away from the Yamaji ones. Nevertheless, one can suppress the Fermi points by aligning the field along an Yamaji angle and this should suppress the associated axial anomaly. As experimentally seen, the suppression of the Fermi points suppresses the negative magnetoresistivity, indicating that the axial anomaly is responsible for it.

In summary, in very clean layered metals we have uncovered a very clear correlation between the existence of Fermi points in a one-dimensional dispersion and the observation of an anomalous negative magnetoresistivity. The suppression of these points leads to the disappearance of this effect. This indicates that the axial anomaly and related negative magnetoresistivity would not be contingent on the existence of an underlying three-dimensional Dirac/Weyl dispersion. Instead, our study in PdCoO_2_, PtCoO_2_ and Sr_2_RuO_4_, which are clean metals with no Dirac/Weyl dispersion at zero magnetic field, indicates that the axial anomaly and its effects could be a generic feature of metal(s) near the quantum limit. Nevertheless, the detection of negative magnetoresistivity would depend on the underlying scattering mechanisms, that is, observable only in those compounds that are clean enough to be dominated by elastic forward scattering^18^,^50^. In a generic metal with a high carrier density, it is currently impossible to reach the quantum limit; for the available field strength, many Landau levels would be populated, thus producing a myriad of Fermi points. In this regard, extremely pure layered metals such as (Pd,Pt)CoO_2_ are unique, as by just tilting the magnetic field in the vicinity of the Yamaji angle one can achieve the condition of a single, partially filled Landau level as it would happen at the quantum limit. An explicit analytical calculation of transport lifetime in the presence of axial anomaly due to multiple partially filled Landau levels is a technically challenging task. Therefore at present we do not have a simple analytical formula for describing the observed (*μ*_0_*H*)^−1^ behavior of the negative magnetoresistance along the *c* axis (for magnetic field strengths much smaller than the one required to reach the quantum limit). Nevertheless, the suppression of this negative magnetoresistivity for fields precisely aligned along the Yamaji angles indicates unambiguously that the electronic structure at the Fermi level is at the basis for its underlying mechanism. The observation of (*μ*_0_*H*)^−1^ behavior in the magnetoresistance around the Yamaji angle (when only one partially filled Landau level contributes) gives us the valuable insight that the anomaly induced negative magnetoresistance is quite robust irrespective of the number of partially filled Landau levels. However the determination of a precise functional form for the magnetoresistance in the presence of multiple partially filled Landau levels remains as a technical challenge for theorists. The situation is somewhat analogous to that of the Weyl semi-metals, which are characterized by a number of Weyl points in the first Brillouin zone^37^, and apparently with all Weyl points contributing to its negative longitudinal magnetoresistivity^46^. Hence, our results suggest that the axial anomaly among pairs of chiral Fermi points may play a role in ultra-clean systems even when they are located far from the quantum limit.

Finally, it is noteworthy that negative longitudinal magnetoresistivity is also seen in kish graphite at high fields, which is characterized by ellipsoidal electron- and hole-like FSs, on approaching the quantum limit and before the onset of a many-body instability towards a field-induced insulating density-wave ground state^51^. As discussed in ref. 18, the axial anomaly on approaching the quantum limit may also play a role for the negative magnetoresistivities observed in ZrTe_5_ (ref. 52) and in *α*−(ET)_2_I_3_ (ref. 53), indicating that this concept, which is the basis of our work, is likely to be relevant to a number of physical systems, in particular semi-metals.

## Methods

### Crystal synthesis

Single crystals of PdCoO_2_ were grown by the self-flux method through the following reaction PdCl_2_+2CoO→PdCoO_2_+CoCl_2_ with starting powders of PdCl_2_ (99.999%) and CoO (99.99+%). These powders were ground for for up to 60 min and placed in a quartz tube. The tube was sealed in vacuum and heated up to 930 °C in a horizontal furnace within 2 h and subsequently up to 1,000 °C within 6 h, and then cooled down quickly to 580 °C in 1 or 2 h. The tube is heated up again to 700 °C within 2 h, kept at 700 °C for 40 h and then cooled down to room temperature at 40 °C h^−1^. Single crystals, with sizes of approximately 2.8 × 1.3 × 0.3 mm^3^ were extracted by dissolving out CoCl_2_ with hot ethanol.

### Single-crystal characterization

These were characterized by powder X-ray diffraction, energy dispersive X-ray analysis and electron probe microanalysis. The powder X-ray diffraction pattern indicated no impurity phases. In the crystals measured for this study, electron probe microanalysis indicated that the ratio of Pd to Co is 0.98:1, and that the amount of Cl impurities is <200 p.p.m.

### Experimental setup

Transport measurements were performed by using conventional four-terminal techniques in conjunction with a Physical Properties Measurement System, a 18-T superconducting solenoid and a 35-T resistive magnet, coupled to cryogenic facilities such as ^3^He systems and variable temperature inserts.

## Additional information

**How to cite this article:** Kikugawa, N. *et al*. Interplanar coupling-dependent magnetoresistivity in high-purity layered metals. *Nat. Commun.* 7:10903 doi: 10.1038/ncomms10903 (2016).

## Supplementary Material

Supplementary Information Supplementary Figures 1-4, Supplementary Notes 1-4 and Supplementary References
