# Supplementary material for: Interplanar coupling-dependent magnetoresistivity in high-purity layered metals
Source: Nat Commun. 2016 Mar 29;7:10903. doi: 10.1038/ncomms10903 (PMC4820545; doi:10.1038/ncomms10903)
Supplement: Supplementary Information — Supplementary Figures 1-4, Supplementary Notes 1-4 and Supplementary References [file ncomms10903-s1.pdf]

Supplementary Figures

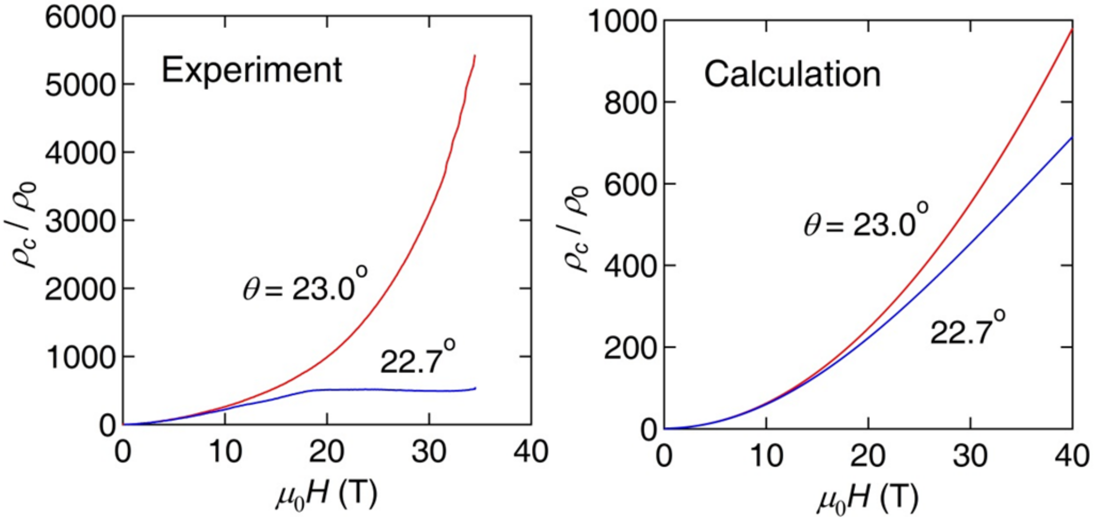

Supplementary Figure 1| Comparison between experimentally observed and calculated magnetoresistive behavior. Left panel: longitudinal inter-layer resistivity ρ_c normalized by ρ_0 as a function of $\mu_0 H$ and for two angles, i.e. the Yamaji angle at θ=23.0^o (red curve) and θ=22.7^o (blue curve). Notice the extremely pronounced positive magnetoresistance, i.e. 500,000 % for fields along the Yamaji angle. Notice how a very small variation in angle leads to a pronounced suppression of the magnetoresistive behavior, or to resistance saturation at the highest fields. Right panel: longitudinal inter-layer resistivity ρ_c normalized by ρ_0 as a function of $\mu_0 H$ according to Boltzmann transport theory for a two-dimensional Fermi surface assuming a single scattering rate τ, see Ref. [1]. Notice how, according to semiclassical transport equations, the magnetoresistance is expected to remain positive and display a H^2-dependence [2] in contrast to what is seen experimentally.

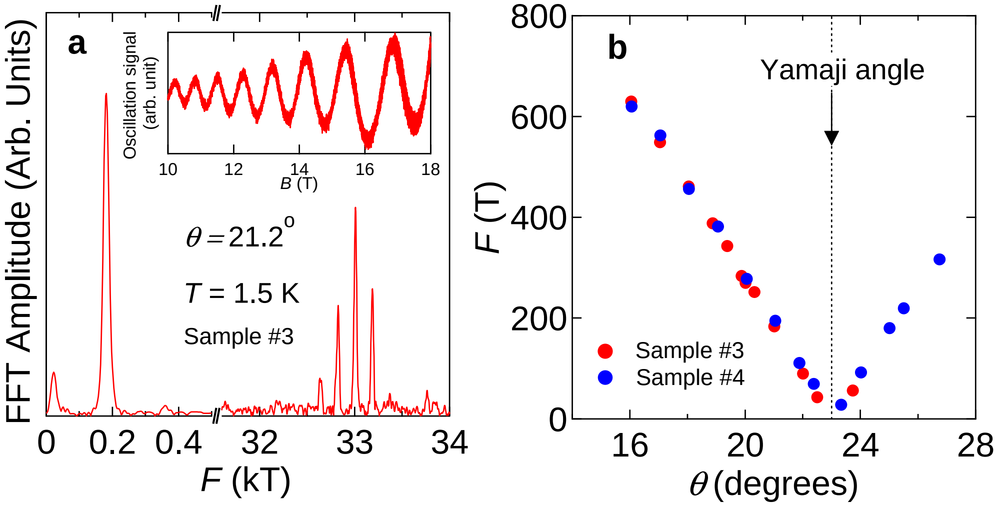

Supplementary Figure 2| Fast Fourier transform of the oscillatory signal and angular dependence of the small frequency. **a** Fast Fourier transform of the oscillatory signal (shown in the inset), or the Shubnikov de Haas-effect, superimposed onto the magnetoresistivity at T = 1.5 K acquired at an angle $\theta = 21.2^{o}$ between the magnetic field and the *c*-axis. At this angle, in addition to the large $F_{\alpha} \cong 30$ kT frequency associated with the cross-sectional area of the hexagonal Fermi surface, one also observes a small frequency $F_{\delta} \cong 180$ T which is attributable to beating between both frequencies associated with FS extremal cross-sectional areas resulting from its corrugation, see Refs. [3,4] **b** Frequency F_{δ} as a function of the angle θ. Notice how $F_{\delta}(\theta)$ is progressively suppressed as $\theta \rightarrow \theta_{n=1} = 23.0^{o}$ which corresponds to a Yamaji angle at which the effective corrugation of the Fermi surface, or the effective inter-layer transfer integral t_c is renormalized to a value approaching zero. Hence, the high field crossover from positive to negative magnetoresistivity as function of the angle, relative to a Yamaji angle, is correlated with the emergence of a finite inter-layer coupling. Red and blue markers depict measurements on two different crystals.

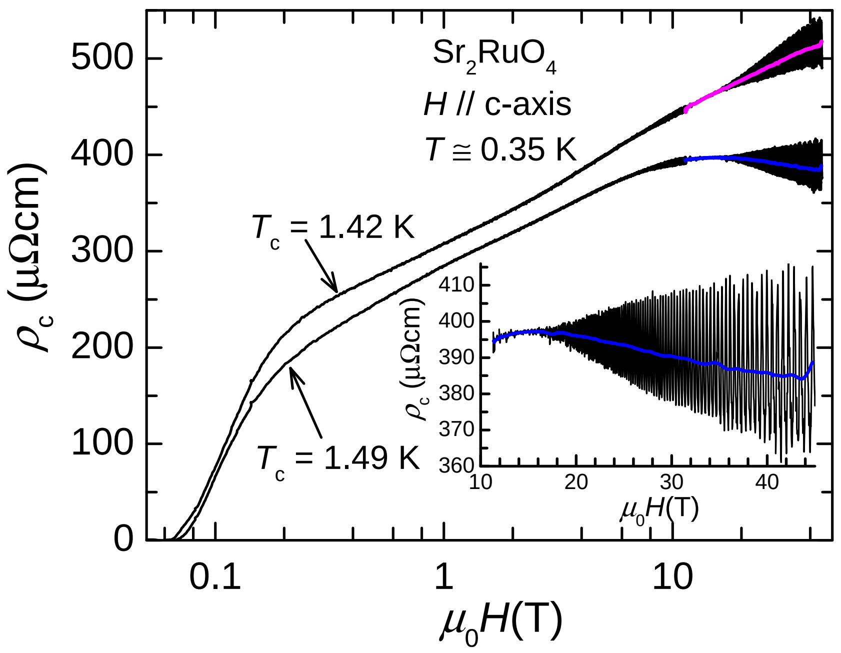

Supplementary Figure 3| Purity dependent longitudinal magnetoresistance in Sr_2RuO_4. ρ_c as a function of $\mu_0 H$ for two Sr_2RuO_4 single-crystals of slightly different purities (or superconducting transition temperatures), and for currents and fields applied along the *c*-axis. Inset: ρ_c as a function of $\mu_0 H$, but in a linear scale, and for the highest purity Sr_2RuO_4 single-crystal. Blue line corresponds to the background magnetoresistivity once the oscillatory component has been suppressed from the original data. Notice how it decreases as a function of H for $H \geq 15$ T, in contrast to what is observed for the lower purity crystal (magenta line).

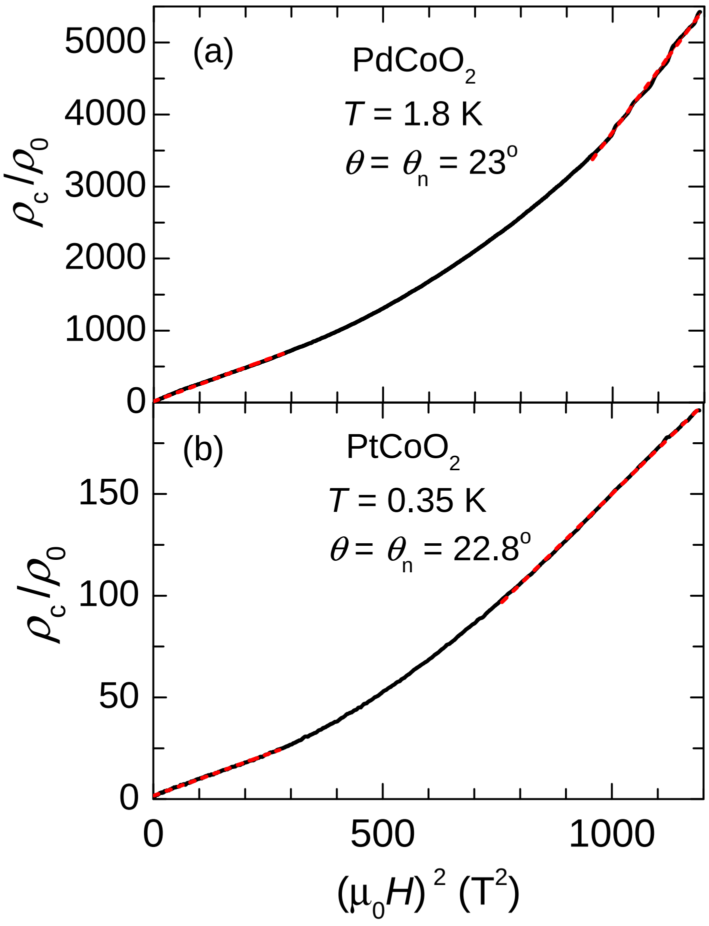

Supplementary Figure 4| Magnetoresistive behavior at the Yamaji angle. a Inter-planar resistivity ρ_c normalized by its zero field value ρ_0 as a function of the square of the field and for a $PdCoO_2$ single-crystal at T = 1.8 K and at the Yamaji angle $\theta = \theta_n = 23^o$. **b** Same as in **a** but for a $PtCoO_2$ single-crystal at T = 0.35 K. In both figures, red dashed lines are linear fits.

Supplementary Notes

Supplementary note 1: Comparison between experiments and semi-classical magnetoresistance calculations

The left panel of Supplementary Fig. 1 above displays ρ_c/ρ_0 when the density of current j || c-axis and the external field H is aligned either along a Yamaji angle or slightly misaligned with respect to it. This is the original data in Fig. 5c in the main text. As seen, at the Yamaji angle the magnetoresistance increases enormously, i.e. by a factor of ~ 500,000 % while a slight misalignment with respect to it leads instead to saturating magnetoresistance at the highest fields. We contrast our experimental observations with what is expected from a semi-classical Boltzmann transport theory developed for explaining the Yamaji-effect in layered or quasi-two-dimensional organic conductors. Please, see Ref. [1] for details concerning this now standard calculation.

According to Ref. [1] the inter-planar conductivity σ_{zz} is given by the following expression:

$$\sigma_{zz} = \sigma_{zz}^{0}\left\{ J_0^2\left(dk_F \tan\theta\right) + \sum_{\nu=1}^{\infty} \frac{2J_\nu^2\left(dk_F \tan\theta\right)}{1+\left(\varpi_c \tau \nu \cos\theta\right)^2} \right\}$$

Where J_0 is zeroth order Bessel function of the first kind, J_ν is the νth -order Bessel function of the first kind, d is the inter-layer spacing, k_F the Fermi vector, $\omega_c = eH/\mu$ (with e being the electron charge and μ the carrier effective mass) and θ is the angle between the magnetic field and the c-axis. For the curves in the right panel of Supplementary Fig. 1 we assumed a single τ as extracted from the in-plane residual resistivity at zero-field with the known value for carrier effective mass, i.e. μ ~1.5 m_0 where m_0 is the free electron mass [3]. We limited the expansion to $\nu = 2$ which still provides a fairly accurate description of the interplanar conductivity in organic metals [1].

As seen, there are marked differences between the experimentally observed and the calculated behavior shown in the right panel of Supplementary Fig. 1. Notice how at the Yamaji angle the experimental magnetoresistivity reaches ~550000 % at H = 35 T, while the calculated would only one reach ~100000 %. Furthermore, the relevant observation here is that for fields misaligned by just 0.3^{o} with respect to the Yamaji angle, the experimentally measured magnetoresistance is observed to saturate, indicating the presence of a second, competing magnetoresistive mechanism. This contrasts markedly with the calculated semiclassical behavior (blue line in the right panel of Supplementary Fig. 1) which would yield a magneto resistance that increases quadratically in field.

Supplementary note 2: Slow Shubnikov de Haas Oscillations for currents along the inter-layer direction.

To more clearly expose the origin of the oscillations at small frequencies, in Supplementary Fig. 2a we display the fast Fourier transform (FFT) of the oscillatory signal superimposed onto $\rho_c(H)$, acquired at an angle θ = 21.2^{o} and at T = 1.5 K. At this angle, one observes a clear frequency $F_\delta \simeq$ 0.18 kT in addition to an ensemble of higher frequencies

centered around 33 kT corresponding to the Fermi surface extremal-cross sectional areas, i.e. F_α and F_β [3], and to interference effects, e.g. $F_\alpha + F_\delta$, etc. Their origin will be the subject of future studies. Supplementary Fig. 2b shows F_δ as a function of the angle θ on a narrow angular range around $\theta_n = 1$. As seen, $F_\delta \rightarrow 0$ as $\theta \rightarrow \theta_n$=1 which clearly demonstrates that i) F_ is associated with the corrugation of the Fermi surface and ii) that this corrugation (in first approximation given by t_c) *vanishes* at θ_n. This is a very clear demonstration for the Yamaji-effect and correlates unambiguously, the negative magnetoresistivity with the emergence of a finite inter-planar coupling or with dispersive Landau levels.

Supplementary note 3: Purity dependent negative magnetoresistivity in Sr_2RuO_4.

Supplementary Figure 3 displays the longitudinal magnetoresistivity ρ_c for fields along the *c*-axis for two Sr_2RuO_4 single crystals having slightly different levels of impurities and hence superconducting transition temperatures (T_c) [5]. The first crystal displays T_c = 1.42 K, and as seen, for fields between its superconducting upper critical field H_{c2}^c ~ 0.15 T and a maximum value of H = 45 T, the inter-planar magnetoresistivity increases by a factor slightly higher than 2 showing no signs of saturation.

The magnitude of this effect bears some resemblance with what was observed for the longitudinal magnetoresistance of elemental Al and which was ascribed to the role of small angle scattering by phonons [2]. In contrast to Al, Sr_2RuO_4 is a correlated two-dimensional Fermi liquid characterized by "quasi-antiferromagnetic" spin fluctuations and concomitant spin-flip scattering [6]. It is unclear if, or how these would contribute to its longitudinal magnetoresistivity or even if they would be affected by a magnetic field. The important observation is that a cleaner sample, i.e., characterized by a T_c = 1.49 K, shows a distinct behavior at the highest fields which is illustrated by the onset of Fig. S4. For this sample, ρ_c increases with field, saturates and, as indicated by the blue line which depicts the background magnetoresistivity after subtraction of the oscillatory component, it subsequently decreases for fields beyond 18 T. Hence, with the suppression of impurity scattering a hitherto unreported negative longitudinal magnetoresistivity is also observed in the cleanest Sr_2RuO_4 single-crystals demonstrating the universality of the effect previously described for the langasites. However, in Sr_2RuO_4 this effect is observed only for angles within 10° away from the *c*-axis. This compound is characterized by three corrugated cylindrical Fermi surface sheets, each leading to a distinct set of Yamaji-angles, making it impossible to completely suppress the inter-planar coupling at a specific angle.

Supplementary note 4: Magnetoresistive behavior at the Yamaji angle

As we discussed in the main text, the Yamaji angle is the angle at which all electronic orbits on a cylindrical Fermi surface end up acquiring the same cross-sectional area. Hence, at the Yamaji angle the Fermi surface of a two-dimensional electronic system effectively loses its corrugation which is responsible for the distribution of cross-sectional areas observed at all the

other angles. Effectively the system acts as if the inter-planar transfer integral vanished at this angle leading to sharp spikes in the inter-planar resistivity.

As seen in the main text, and in contrast to what is observed for angles away from the Yamaji angle, we only observe a positive but very pronounced magnetoresistivity. We find that this magnetoresistivity cannot be described through a single power-law over the entire field range. However, as seen in Supplementary Fig. 4, at lower fields (or below 15 T), for both $PdCoO_2$ and $PtCoO_2$, the magnetoresistivity increases quadratically in field as is usually seen in clean, coherent metals [1,2,7]. For both compounds it seems to recover the H^2 dependence at higher fields although it is characterized by a more pronounced slope, after the resistivity undergoes a crossover like, non-quadratic in field, region.

According to the Kohler's rule $\Delta\rho/\rho_0 \cong (\mu_0 H/\rho_0)^2$, hence an increase in the slope of the quadratic in field dependence would indicate a decrease in the strength of scattering processes by impurities. The precise reason for this remains unclear at the moment. Perhaps at higher fields the increase in the degeneracy of the Landau levels, or in the concomitant density of states at the Fermi level, could contribute to a more effective Thomas-Fermi screening of the charged impurities. Notice, that for fields along θ_n all orbits are closed while for fields along [1-10]-direction the electronic orbits are composed of a collection of open orbits along the axis of the cylindrical Fermi surface, and of closed orbits exploring the corrugation of the Fermi surface. Therefore, these distinct sets of orbits for either field orientation explore different paths on the Fermi surface and are likely to be exposed to distinct scattering cross-sections. Consequently, they would be expected to provide distinct contributions to the magnetoresistivity.

Supplementary References

[1] Yagi, R., Iye Y., Osada, T., & Kagoshima, S. Semiclassical Interpretation of the Angular-Dependent Oscillatory Magnetoresistance in Quasi-Two-Dimensional Systems. *J. Phys. Soc. Jpn.* **59**, 3069-3072 (1990).

[2] Pippard, A. B. *Magnetoresistance in Metals*. Cambridge Studies in Low temperature Physics 2, Cambridge University Press (1989).

[3] Hicks, C. W., et al. Quantum Oscillations and High Carrier Mobility in the Delafossite $PdCoO_2$. *Phys. Rev. Lett.* **109**, 11640 (2012).

[4] Kartsovnik, M. V., Grigoriev, P. D., Biberacher, W., Kushch, N. D. & Wyder, P. Slow Oscillations of Magnetoresistance in Quasi-Two-Dimensional Metals. *Phys. Rev. Lett.* **89**, 126802 (2002).

[5] Mackenzie, A. P., Haselwimmer, R. K. W., Tyler, A. W., Lonzarich, G. G., Mori, Y., Nishizaki, S. & Maeno, Y. Extremely strong dependence of superconductivity on disorder in Sr^2RuO_4. *Phys. Rev. Lett.* **80**, 161-164 (1998).

[6] Braden, M., Steffens, P., Sidis, Y., Kulda, J., Bourges, P., Hayden, S., Kikugawa, N. & Maeno, Y. Anisotropy of the Incommensurate Fluctuations in Sr_2RuO_4: A Study with Polarized Neutrons. *Phys. Rev. Lett.* **92**, 097402 (2004).

[7] Takatsu, H. et al. Extremely Large Magnetoresistance in the Nonmagnetic Metal $PdCoO_2$. *Phys. Rev. Lett.* **111**, 056601 (2013).
